# Policies out of sync – are healthy ageing agendas fit for the future?

**DOI:** 10.1093/eurpub/ckac129.564

**Published:** 2022-10-25

**Authors:** A Mavrodaris, L Lafortune, C Brayne

**Affiliations:** 1 University of Cambridge, Cambridge Public Health, Cambridge, UK; 2 Department of Health and Social Care, Office for Health Improvement and Disparities, London, UK

## Abstract

**Background:**

In both scale and impact, population ageing has far reaching implications for our planet, not least as a major driver of population growth and the ever-increasing human demands on natural resources and ecosystems. This fundamentally impacts sustainable development efforts to eradicate poverty, achieve food security, build inclusive and resilient communities, and ensure sustainable consumption. The overarching connections between global ageing and sustainability are clear: a focus on sustainable healthy ageing is fundamental to a healthy planet. Our responses to date have however largely been disconnected. To progress this dual agenda, our work aims to i) assess whether current national/international strategies addressing healthy ageing include a strategic focus on sustainability; ii) present the evidence for such alignments; and iii) develop a framework of sustainable actions and aligned policy.

**Methods:**

A mixed-methods approach using content and applied thematic analysis was utilised to examine strategy documents, and develop an analytical framework derived from relevant theory to guide quantitative and qualitative analysis of the resultant data. Evidence themes were developed iteratively during analytical phases. Findings informed the development of the framework.

**Results:**

We identified and analysed 36 strategies published from 2000 to 2021 containing over 600 wide-ranging policies. No strategies and only a minority of policies included a strategic sustainability focus. A larger subset made reference to links between ageing and sustainability or environmental elements yet these were largely theoretical and not carried through in the key strategic approaches or resulting polices.

**Conclusions:**

This work provides valuable insights into strategic approaches to foster sustainable healthy ageing and identifies levers for greater alignment and sustainable action. The recently declared 2021-30 UN Decade of Healthy Ageing provides an ideal platform for action.

**Key messages:**

• While the evidence for strong alignment is unequivocal, global healthy ageing and sustainability agendas are largely disconnected.

• By strengthening the links between healthy ageing and sustainability agendas, stakeholders across sectors can reinforce and design approaches that meet human needs while protecting our planet.

